# Improvements on GPS Location Cluster Analysis for the Prediction of Large Carnivore Feeding Activities: Ground-Truth Detection Probability and Inclusion of Activity Sensor Measures

**DOI:** 10.1371/journal.pone.0138915

**Published:** 2015-09-23

**Authors:** Kevin A. Blecha, Mat W. Alldredge

**Affiliations:** 1 Graduate Degree Program in Ecology, Colorado State University, Fort Collins, Colorado, United States of America; 2 Mammals Research Division, Colorado Parks and Wildlife, Fort Collins, Colorado, United States of America; University of Queensland, AUSTRALIA

## Abstract

Animal space use studies using GPS collar technology are increasingly incorporating behavior based analysis of spatio-temporal data in order to expand inferences of resource use. GPS location cluster analysis is one such technique applied to large carnivores to identify the timing and location of feeding events. For logistical and financial reasons, researchers often implement predictive models for identifying these events. We present two separate improvements for predictive models that future practitioners can implement. Thus far, feeding prediction models have incorporated a small range of covariates, usually limited to spatio-temporal characteristics of the GPS data. Using GPS collared cougar (*Puma concolor*) we include activity sensor data as an additional covariate to increase prediction performance of feeding presence/absence. Integral to the predictive modeling of feeding events is a ground-truthing component, in which GPS location clusters are visited by human observers to confirm the presence or absence of feeding remains. Failing to account for sources of ground-truthing false-absences can bias the number of predicted feeding events to be low. Thus we account for some ground-truthing error sources directly in the model with covariates and when applying model predictions. Accounting for these errors resulted in a 10% increase in the number of clusters predicted to be feeding events. Using a double-observer design, we show that the ground-truthing false-absence rate is relatively low (4%) using a search delay of 2–60 days. Overall, we provide two separate improvements to the GPS cluster analysis techniques that can be expanded upon and implemented in future studies interested in identifying feeding behaviors of large carnivores.

## Introduction

The advent of GPS telemetry has transformed the ability of ecologists to systematically quantify animal movement, landscape use, and specific behaviors such as feeding. For large carnivores, GPS telemetry has been used for over a decade to identify feeding events by assessing the proximity of GPS locations to one another in space and time through GPS location cluster analysis [1]. While other methods such as snow tracking [2], intensive VHF telemetry [3,4] or even live observations [5] may be used to identify feeding events in certain systems, GPS location cluster techniques can be applied in an efficient systematic manner to quantify when, what, and where a predator kills. Initially, kill rates (e.g., how often a predator kills; [1]) appeared to be the main parameter sought after using cluster analysis. Studies have since expanded to utilizing the locational aspect of the feeding event data. This “where” component can then be used as input into behavior specific resource selection functions that examine usage with respect to the landscape characteristics [6,7], spatial availability of prey [8–10] or disturbance risks [11]. Combining the “when” and “where” helps provide a mechanistic understanding of the biological processes at hand.

The cluster analysis technique [1] entails: 1) collaring a focal animal with a GPS collar, 2) logging GPS location data on the collar and retrieving the data, 3) identifying potential feeding events from grouped locations of data (GPS clusters), and 4) ground-truthing clusters by field personnel to assess whether the GPS location cluster represents a kill made by the focal collared animal. From here, the characteristics of the animal consumed (species, sex, age, etc.) may be determined.

Data collected in ground-truthing efforts can then be used in predator studies in one of two ways. Assuming that all clusters identified are ground-truthed, the presence or absence of prey remains can be used directly [12,13]. However, confirming presence/absence of prey at all clusters in large studies or certain systems is impractical. Therefore, a sample of clusters, representative of a wide variety of conditions, can be ground-truthed [14–16]. Presence/absence data of prey remains can then be used as a binary response variable into a prediction model, which is the focus of this paper. Modeling usually involves relating a binary response variable with a logit link function to a design matrix of spatio-temporal predictor variables, such as the Euclidean distance of GPS locations to one another within the cluster, or the amount of time spent by the animal at the cluster [1,12,13]. While many clever spatio-temporal covariates are likely tested in the models, many ultimately end up collinear with each other. Such as the case when the number of nights spent at a cluster is correlated with the duration (hours) spent at a cluster [13]. Incorporating other sources of bio-telemetry data collected independently from GPS location data may improve model prediction accuracy.

Activity data is one such data source yielding information independent of GPS spatio-temporal data. Activity sensor data is increasingly used to give basic information on physical behavior states (i.e., moving vs. stationary) and to identify feeding behaviors of predators [17–20]. Incorporating activity sensor data as an additional covariate(s) alongside spatio-temporal covariates in a binomial regression model to predict feeding behaviors would be the next logical step for large carnivore feeding studies. McClintock et al. [21] found that by incorporating activity and GPS location data recorded on a marine mammalian predator improved typical behavioral state predictions made with GPS locational data alone. While more complex algorithms could be developed to identify periodicity of activity within specified time intervals [17], it seems that simple descriptive statistics of the activity data could be calculated for a temporal window around each GPS location, thus standardizing to match the temporal acquisition rate by the GPS collar. The aggregated data could then be used as another covariate in a typical logistic regression feeding prediction model, potentially improving prediction success. Because the unit of analysis is still the GPS cluster location, this post-hoc integration would enable predicted feeding events to be identifiable spatially, rather than merely events in time as done with earlier accelerometer applications with large terrestrial carnivores [20,22].

Feeding prediction models can be subject to incomplete detection of feeding events, ultimately leading to naïve estimates of feeding event probabilities, which transfers to other commonly sought metrics (i.e., kill rates). One source of detection error is from GPS location acquisition misses [12]. A second source may be from data transmission delays, where techniques used to retrieve GPS locations from collars remotely, via satellite data uplink, may not operate with perfect success. If ground-truthing visits are done using the individual GPS locations to guide the search effort, a spatially dispersed cluster may be searched inadequately in the case of missing GPS locations. Full recovery of logged GPS locations can eventually be conducted with other methods (i.e., removal of collar), but this is usually done post ground-truthing. Because modeling efforts utilize all logged GPS locations as input, a discontinuity can exist between the data used for ground-truthing the clusters and the data used for the prediction model. The third source of error is also related to ground-truthing, where the amount of time between the formation of a cluster by a predator and the visitation by an observer has a negative influence on the probability a cluster is determined as a feeding event [13,23,24].

While conducting studies on cougar (*Puma concolor*) feeding habits (i.e., prey composition, kill rate, and resource selection analysis) using GPS cluster techniques in the Front Range of Colorado, we develop two separate improvements to feeding site prediction models. First, we build prediction models augmented with biotelemetry activity data and compare the candidate models to simpler models in terms of model fit and prediction performance. Second, we address various issues regarding detectability of prey remains while ground-truthing. We build feeding site prediction models with and without various covariates concerning detectability and compare the candidate models in terms of model fit, prediction performance, and predicted feeding event counts. Additionally, a double observer study was conducted to assess the ability of ground-truthing observers to classify presence or absence of prey remains up to 60 days after initiation of the cluster by a cougar.

## Methods

### Data Collection and Ethics Statement

Cougars were captured with the aid of trained hounds, baited cage traps/foot snares, or by using known active feeding sites of collared or un-collared cougars. Baiting involved placing an ungulate carcass at targeted locations and monitoring with remote cameras. Cage traps and foot snares were only deployed once a cougar was found to be actively visiting a carcass or when found at a carcass killed by the cougar. This allowed a capture crew to monitor trap motions at a nearby location continuously via a VHF radio transmitter system. Cougars were chemically immobilized with medetomidine-ketamine, which was then reversed with atipamezole. Cougars responded within 2–5 minutes of administration of immobilization and reversal agents. No cougars were injured during capture. Cursory examination of GPS location data indicated all cougars returning to normal activities within 12–24 hours of capture. Animal capture and handling was conducted under the strict guidelines set forth by a protocol approved by the Colorado Parks and Wildlife Institutional Animal Care and Use Committee (USDA registration # 84-R0045: protocol # 16–2008). Cougar capture and ground-truthing visitations occasionally occurred on privately owned lands in which permission to access was obtained by field crews.

Cougars were fitted with Vectronic GPS collars (Model: GPS-Plus, Vectronic Aerospace GmbH, Berlin, Germany) programmed to record 7 GPS locations per day (every 3 hours during the night and every 4 hours during the day: 02:00, 05:00, 08:00, 12:00, 16:00, 19:00, 21:00). Collars featured Global Star or Iridium satellite GPS location downloading, basic flash memory data storage, and a two-axis accelerometer-based activity sensor. For each axis of the sensor, activity data was logged as a single continuous measure (ranging 0–255) accumulating for every 288 second block of time. Collars were programmed to upload GPS locations to an email account in near real-time. Activity data, stored on-board, was retrieved by downloading on the ground through VHF transmission or upon removal/replacement of the collar.

Ground-truthing visitations were implemented with a randomized sampling scheme [14–16], with a three year study period stratified into 36 monthly intervals (January 1, 2010 –December 31, 2012. At the end of each monthly interval we applied a clustering algorithm script to the GPS collar locations for each individual cougar. Based on spatial and temporal characteristics, the script classified all GPS locations downloaded via satellite into one of five unique selection sets (*S1*, *S2*, *S3*, *S4*, and *S5* types) [25]. However, this paper is only concerned with the *S1* and *S2* cluster types. *S1* clusters are any group of at least 2 locations within 200 meters and within 4 days of each other, similar to many other studies [1,11,12]. *S2* cluster types represented two GPS locations separated by 200–500 m, but with a scheduled GPS location missing (collar failed to record) in between the two GPS locations [25].

**Fig 1:**
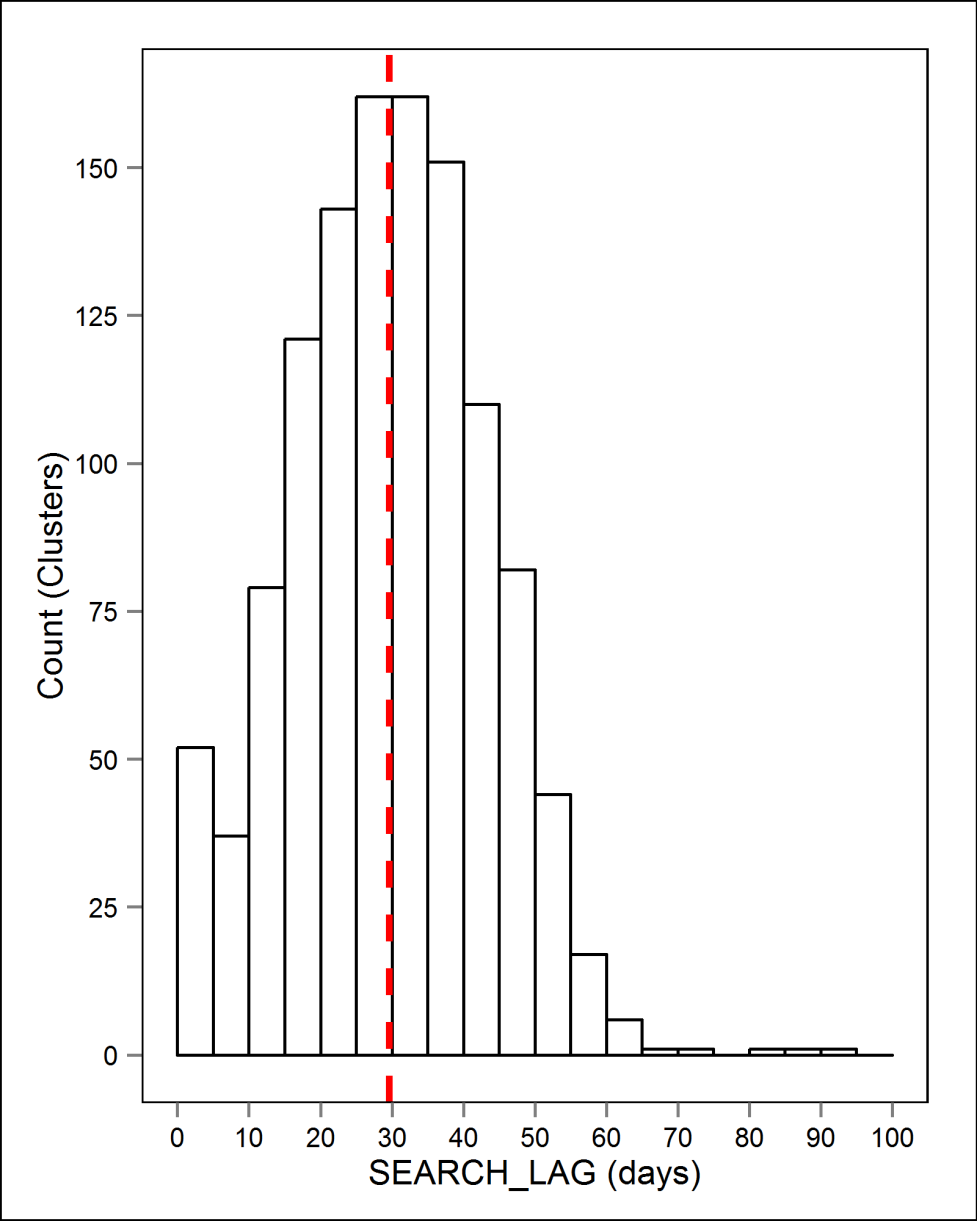
Histogram of Search Lag Times. The distribution of time (SEARCH_LAG) between the initiation of a kill by cougar and the visitation by a ground-truth observer for 1171 unique cluster visits. Red dashed line indicates the distribution mean.

Conducting ground-truthing visits throughout each of the 36 monthly strata ensured that a large temporal continuum of conditions (i.e. changes in season, weather, and human activities) were represented. Random numbers were assigned to clusters within each strata, in which the top two random *S1* clusters and the top *S2* cluster were visited by a ground-truthing observer. In the rare case that technicians did not have access to the cluster site (i.e., permission denied by landowner or land manager), the cluster with the next highest random number was visited. Clusters were ground-truthed throughout the monthly period following the month the constituent GPS locations occurred in, allowing sampled clusters to be visited a mean of 29.6 days (range = 0–93, stdev = 13.64) post initiation by the cougar (Fig 1). This monthly sampling period allowed: 1) the cougar to vacate the site in most instances, thus reducing researcher disturbances, 2) a number of potential feeding events to have occurred, thus providing a reasonable population of clusters to be sampled from. In the field, ground-truthing observers were directed to not prioritize within the monthly period by any particular cluster type, cougar, or habitat unless inclement winter weather hindered access to a specific area. Observers were assigned to cougars and geographic sub-regions on a monthly rotation to account for differences in observer abilities. Novice observers were trained and accompanied by an experienced observer for 2–4 weeks, prior to ground-truthing on their own, to aid development of a prey remains search image.

A radius of 50 meters was exhaustively searched for each GPS location composing the sampled cluster site until prey remains were discovered. However, all spatially outlying GPS locations were eventually visited. Clusters were classified with a binary presence/absence indicator of a feeding event. In the case of a feeding event, prey species, age, and sex were identified. Body mass estimates were assigned based on Armstrong et al. [26]. It is assumed that the presence of an animal carcass remains indicated feeding activities, regardless if cause of death was from the focal cougar. Body mass for mule deer (*Odocoileus hemionus*; the most common prey species found at clusters) less than 1 year of age were calculated [27] assuming a birthdate of June 26.

### Feeding Prediction Model

Prior to statistical modeling, but after all GPS location data stored on collars had been retrieved, the clustering algorithm was rerun at the end of the study to incorporate any data un-retrievable via satellite during monthly investigation intervals. GPS locations recorded within 7 days of capture or locations spatially and temporally associated with known natal dens were truncated. Generalized linear models (program R, v.3.1.1) were used with a logit link function to model the binary response of the presence (1) or absence (2) of feeding remains at a cluster as a function of covariates. These covariates were grouped into three major classes: spatio-temporal characteristics of the GPS locations constituting the cluster, activity sensor measures, and ground-truthing error attributes.

GPS location spatio-temporal characteristics take advantage of the particularly long handling times of feeding cougars. However, cougars also exhibit movement behaviors to and away from clusters containing larger prey items over the course of consumption. The number of GPS locations (POSCOUNT) collected at the cluster is a proxy for the amount of time potentially consuming the carcass. Clusters enduring more than a 24 hour period (i.e. > 1 night) were characterized by a binary variable (TIMEBIN24). Most feeding activities take place during the night, while day-time resting activities often occur outside of the cluster defined spatial extent (the 200 m radius), thus the proportion of GPS locations in the cluster that were collected during the night time was a covariate (NIGHTPROP). An interaction between POSCOUNT and NIGHTPROP was included to help distinguish between repeat usage of day-bed sites and that of feeding sites. Spatial dispersion of GPS locations within a cluster (CENTER) was measured as the average distance from the geometric center of each cluster to its constituent GPS locations. However, allowing CENTER to interact with other covariates may allow more dispersed clusters (e.g., clusters with bimodal point dispersion) to be indicative of feeding in certain cases.

Activity sensor data was used to derive metrics that may enhance predictions by scrutinizing between resting and feeding activities. The sensor’s X-axis measures the relative amount of forward-backward movements, while the Y-axis measures the relative amount of side to side and head-rolling movements. ACCX was calculated as the mean of all 5 minute activity intervals recorded on the x-axis within a 1.5 hour window of all locations constituting the cluster. This measure was recorded for the y-axis activity sensor as well (ACCY). ACCX and ACCY measures showed significant positive correlation (Pearson corr. coef. = 0.92). Despite this, we retained both measures, but never combined ACCX and ACCY main effects in the same model. Subtracting the ACCY from the ACCX value (ACCXYDIFF), an independent measure harnessing information from the ACCX and ACCY measures was obtained. Specifically, ACCXYDIFF was the average difference of the two axis within a 1.5 hour window. Exploratory examination of the data suggested ACCXYDIFF yielding more positive vs. more negative values at confirmed feeding events. Activity and GPS spatio-temporal characteristics would be expected to have interacting effects, especially in the case of shorter feeding bouts on smaller prey items. Feeding events on smaller prey items would normally be predicted as having a lower predicted feeding probability based on cluster length (i.e., POSCOUNT), but not necessarily so when combined with relatively high activity measures.

Ground-truthing error attributes are nuisance variables that potentially underlie a false-absence classification of the cluster by a human observer. The GPS fix acquisition success rate (FIXRATE) was calculated on a moving window for a 96 hour time period for each GPS location logged, which then was averaged among all GPS locations in the cluster. The proportion of locations downloaded successfully via satellite linkage (FIELDPROP) was calculated for each cluster. We predicted that a lower FIELDPROP and FIXRATE values would translate to observers having a slightly mis-focused investigation spatially, as an observer’s search effort is normally greater near the known GPS locations. Since clusters were ground-truthed anywhere from 1–60 days post feeding, the search lag time (SEARCH_LAG) was used as a continuous variable to characterize an increasing false negative classification rate associated with carcass degradation or dispersion by scavengers over time. Tambling et al. [23] found that the percentage of clusters ground-truthed containing kills declined within the first four weeks, but was constant within the following 16 weeks. Other studies were successful at locating at least some prey remains with time lags of 6–12 months [1,23]. Therefore we tested SEARCH_LAG as a negative exponential decay function in addition to a simple linear term. The season (SEAS) (SUM: Jun 1 –Sep 30, FAL: Oct 1 –Jan 15, WIN: Jan 16 –May 31), was used as a proxy for general seasonal differences between carcass decay rates associated with weather, as well as potential seasonal differences in the types of prey cougars use. Smaller prey would be assumed to degrade and/or be displaced away from the feeding site quicker than larger prey.

### Model Selection and Performance

Candidate models were built using all combinations of the spatio-temporal attributes, activity sensor, ground-truthing errors, and season covariates as individual components. We considered quadratic terms, log transformations, and the afore-mentioned two-way interactions. AIC model selection techniques were used to select the top parsimonious model [28]. For simplification of displaying the model selection results, this top model was then broken down into 16 separate models based on the 4 covariate grouping components: spatio-temporal attributes, activity sensor, ground-truthing error, and season covariates (“SpTemp”, “Acvty”, “GrndTru”, and “Seas” respectively). Interaction terms in the top model sharing covariates between two components were not allowed to occur unless both components were present. These 16 models were then compared using various performance measures of model prediction accuracy. Predictions of each model were applied back to the input dataset of investigated clusters to derive a predicted probability. The predicted probability was discretized into feeding remain presence/absence by choosing a cut-off value. This cut-off value for each model was chosen to balance sensitivity (true-positive rate) and specificity (true-negative rate) [12,29]. Receiver Operator Characteristics (ROC) analyses (R package: ‘ROCR’) were then carried out to calculate the area under the curve (AUC), which ranged from 0–1.0. An AUC of 0.5 would indicate that the model does not predict any better than random chance, while a value of 1.0 indicates perfect discrimination. Theoretically, one can build a model that has perfect prediction performance when validating models with the same set of clusters used in model construction. However, predictions will only be applicable to that particular set of input clusters. To protect against this potential over-fitting, the average AUC measure using K-fold (k = 20 hold out sets) was calculated [12,30]. A K-fold cross validation AUC measure that exceeds a simple direct AUC measure conducted on the same model may be evidence of over-fitting and thus warranting a model with a simpler structure. In this case, the model term with the least contribution to a greater log-likelihood was dropped.

After model performance measures were calculated, a final reapplication of the model to the entire dataset (all clusters visited and not visited) was conducted to reflect the final feeding event count when ground-truthing errors were and were not accounted for. Values for the variables SEARCH_LAG and FIELDPROP would not be present for unvisited sites and thus were treated in two ways. When accounting for ground-truthing errors, it is recognized that these are nuisance variables reflective of researcher capabilities, and not actually the animal’s behavior or GPS collar data. Thus, values for SEARCH_LAG were set to 0 days and the FIELDPROP to 1.0 in the entire dataset. When not accounting for ground-truthing errors, SEARCH_LAG and FIELDPROP values were set to the mean values of what was obtained when ground-truthing (29.1 days and 0.913 respectively) for the whole dataset. A simple enumeration of the predicted feeding events and 95% Wald’s confidence intervals (1.96*Std Error), associated with the proportion of total feeding events, were produced for each model.

### Double Observer Experiment

To experimentally examine the false-absence rate induced by a visitation delay, a subset of GPS location clusters, retrieved via satellite download link, were visited by an independent investigator during or soon after the cougar initiated prey handling activities. These visitations were termed as “initial investigations”. Using the near-real time data downloaded via satellite, this investigation occurred 0.25 days (~6 hours) from the apparent start of the cluster to within 2 days following the termination of the cluster. The automated cluster algorithm [25] was designed to run on larger strings of GPS location data (i.e., monthly intervals), and not for collar data downloaded in real-time. Therefore real-time data was manually assessed (visualized using simple mapping software) for locations where a cougar logged at least 2 GPS locations occurring within approximately 50 m in a 24 hour period. While conducting initial investigations, care was taken to not disturb the cougar if still present; thus a full search was not always possible. A subset of clusters visited during the standard monthly investigation period occasionally coincided as also being the randomly sampled cluster in which a ground-truthing visit was made in the standard 2–60 day interval. This subset of clusters, visited by two separate observers (one at the initial cluster visitation and one at the standard visitation), were the basis for the experimental analysis. This seemingly haphazard sampling of initial clusters occurred because intentionally directing a second observer to visit clusters in which an initial investigation was already made, may cause an observer to put forth a more prudent search unrepresentative of the other standard investigations search effort. Thus the second observer (the one conducting the standard visitation) was completely blind to whether an initial search was even made. Besides allowing an independent classification, it resulted in the second observer’s mean search time (31.7 days) to approximate that of all sites visited under the standard monthly visitation scheme of 29.6 days. Simple calculations were made between the standard and initial investigations to determine the concordance of classification (proportion of clusters matched). Since smaller prey items may degrade quicker, a simple small and large prey classification was given for each prey item found. Small and large prey items were distinguished with cutoff mass of 23.7 kg, which corresponds to mule deer fawn size at the transition from the summer to fall seasons (October 1).

## Results

For the three year study period, 33 collared cougars (24 females and 9 males) were monitored for a mean 495 days (range: 46–1000) per subject, resulting in the retrieval of 90,950 useable GPS locations. Using this as clustering algorithm input, 12,096 clusters were classified. GPS acquisition success rates calculated over unique combinations of individual cougars and monthly period were 0.808 (std dev = 0.162, range = 0.19–1.0). Real-time downloads of GPS location data were successful for 92% of the locations stored on the collar. Approximately 5.6 million activity sensor records for the x- and y-axis, collected in 288 second intervals were retrieved, and then aggregated into 90,950 records corresponding to the number of GPS locations. A total of 1,171 clusters were ground-truthed. Prey species found ranged in size from 1–450 kg with 38.2% being small prey items (i.e., < 20 kg).

Examining all combinations of the predictor variables, the most parsimonious model contained spatio-temporal, activity, ground-truthing, and season terms (Table 1). Model selection measures and coefficient estimates for the set of candidate models holding a cumulative 95% of the AICc weight are given in Table 1. Collinearity between any two predictor variables never exceeded a Pearson’s correlation of 0.34. Comparing models reduced to their respective covariate grouping components, the top model (SpTemp + Acvty + GrndTru + Seas) held 0.919 of the AICc weight, while the model without the ground-truthing terms (SpTemp + Acvty + Seas) held most of the remaining (0.08) AICc weight (Table 2).

**Table 1:** Model selection table and coefficient estimates (non-standardized) of the candidate model set holding a cumulative AICc weight of 0.95.

	Spatio-temporal Covariates (SpTemp)					Activity Covariates (Acvty)					Ground-truthing Error Covariates (GrndTru)							
(Intercept)	CENTER	CENTER^2^	log POSCOUNT	NIGHTPROP	log POSCOUNT X NIGHTPROP	ACCX	ACCX^2^	ACCXYDIFF	ACCX X CENTER	ACCXYDIFF X log POSCOUNT	FIELDPROP	FIELDPROP^2^	SEARCH_LAG	SUM	df	logLik	ΔAICc	AICc weight
-9.074	-0.016	0.00013	1.521	-1.515	2.155	0.148	-0.00071	-0.013	-0.00061	0.115	7.989	-4.664	-0.018	+	15	-327.5	0.00	0.297
-6.817	-0.016	0.00013	1.542	-1.561	2.189	0.147	-0.00070	-0.011	-0.00062	0.114	1.114	-	-0.019	+	14	-328.7	0.16	0.274
-5.774	-0.016	0.00013	1.500	-1.447	2.131	0.149	-0.00072	-0.005	-0.00061	0.105	-	-	-0.019	+	13	-330.2	1.12	0.170
-9.759	-0.017	0.00013	1.555	-1.431	2.110	0.145	-0.00069	-0.007	-0.00059	0.107	8.242	-4.774	-	+	14	-330.2	3.18	0.061
-7.454	-0.016	0.00013	1.580	-1.473	2.138	0.143	-0.00067	-0.005	-0.00059	0.107	1.202	-	-	+	13	-331.3	3.45	0.053
-8.862	-0.019	0.00014	1.539	-1.560	2.196	0.151	-0.00072	0.099	-0.00060	-	7.552	-4.491	-0.017	+	14	-331.0	4.87	0.026
-6.353	-0.017	0.00013	1.532	-1.350	2.079	0.146	-0.00070	0.001	-0.00058	0.097	-	-	-	+	12	-333.1	4.87	0.026
-6.685	-0.018	0.00014	1.565	-1.601	2.222	0.149	-0.00071	0.100	-0.00061	-	0.925	-	-0.017	+	13	-332.1	4.94	0.025
-5.827	-0.019	0.00014	1.530	-1.500	2.171	0.151	-0.00073	0.098	-0.00060	-	-	-	-0.018	+	12	-333.1	5.06	0.024

^2^ refers to a quadratic term

X refers to interaction between variables.

**Table 2:** Logistic regression models for predicting feeding presence/absence at GPS cluster sites were compared with AIC model selection, feeding event predictive performance, and predicted number of feeding events. For simplification, models shown are reduced to components based on: Spatio-temporal (SpTemp), Accelerometer activity sensor (Acvty), ground-truthing errors (GrndTru), and calendar season (Seas) covariates.

	Model Selection				Predictive Performance				Predicted Feeding Event Count		
Model Components	Param. Count	Deviance (log Likelihood)	ΔAICc	AICc Weight	Probability Cut-off	AUC	Sensitivity/Specificity	20-fold cross validated AUC	GrndTru Uncorrected	GrndTru Corrected	Prop. of the 12,096 Clusters Identified as Feeding (95% C.I.)
SpTemp* + Acvty^†^ + GrndTru^‡^ + Seas^§^	15	-327.5	0	0.92	0.372	0.893	0.893	0.889	3045	3360	0.278 (0.237–0.344)
SpTemp + Acvty + Seas	12	-333.1	4.9	0.08	0.372	0.891	0.891	0.886	3146	-	0.26 (0.23–0.301)
SpTemp + Acvty + GrndTru	14	-339.4	21.6	0.00	0.367	0.883	0.883	0.877	2944	3200	0.265 (0.225–0.323)
SpTemp + Acvty	11	-344.5	25.8	0.00	0.381	0.883	0.883	0.881	2892	-	0.239 (0.213–0.272)
SpTemp + GrndTru + Seas	10	-412.4	159.5	0.00	0.367	0.840	0.841	0.837	3042	3539	0.293 (0.235–0.394)
SpTemp + Seas	7	-417.5	163.6	0.00	0.368	0.833	0.833	0.829	2633	-	0.218 (0.198–0.24)
SpTemp + GrndTru	9	-442.4	217.4	0.00	0.35	0.824	0.825	0.816	2614	2926	0.242 (0.193–0.283)
SpTemp	6	-446.2	218.9	0.00	0.345	0.823	0.823	0.824	2780	-	0.23 (0.196–0.254)
Acvty + GrndTru	7	-548.6	425.9	0.00	0.437	0.816	0.817	0.802	4167	3886	0.321 (0.257–0.398)
Acvty + GrndTru + Seas	8	-548.4	427.3	0.00	0.436	0.818	0.819	0.811	4253	3982	0.329 (0.26–0.415)
Acvty^†^	4	-566.5	455.6	0.00	0.447	0.819	0.819	0.820	3596	-	0.297 (0.263–0.33)
Acvty + Seas	5	-566.1	456.7	0.00	0.444	0.821	0.821	0.815	3528	-	0.292 (0.251–0.332)
GrndTru + Seas	5	-755.6	835.7	0.00	0.395	0.640	0.643	0.626	12096	3896	0.322 (0–1)
GrndTru^‡^	4	-761.1	844.8	0.00	0.388	0.634	0.633	0.631	12096	12096	1 (0–1)
Seas^§^	2	-791.9	902.2	0.00	0.386	0.550	0.419	0.547	3896	-	0.322 (0.322–1)

Each component includes:

*log(POSCOUNT), NIGHTPROP, log(POSCOUNT)*NIGHTPROP, CENTR, CENTR^2^

^†^ACCX_AVG, ACCX_AVG^2^, ACCXYDIFF_AVG

^‡^SEARCH, FIELDPROP, FIELDPROP^2^

^§^SUM (binary)

**Fig 2:**
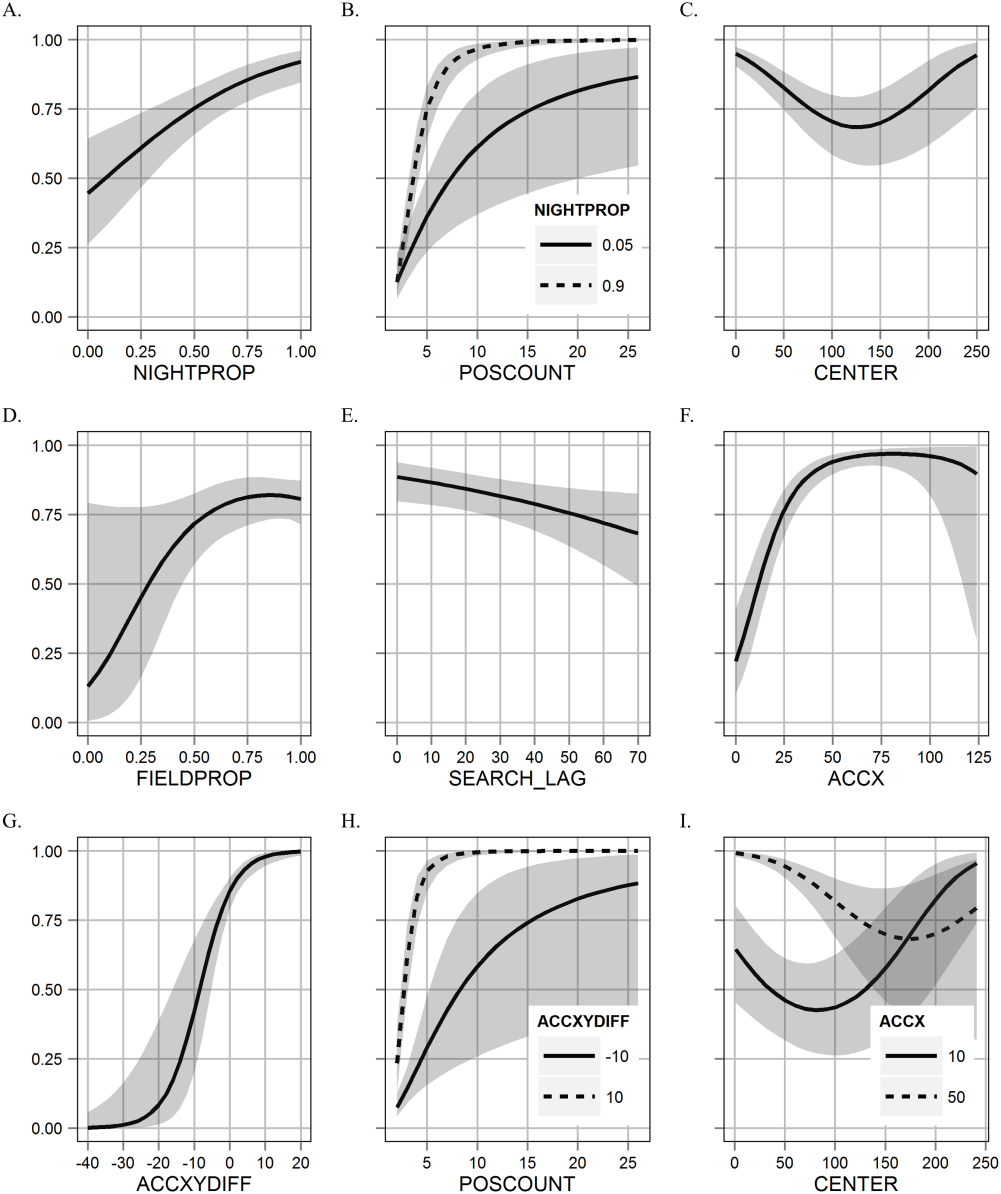
Feeding Event Probability Response Plots. Response plots for the predicted probability (y-axis) of a cluster being a feeding event and 95% CI (gray shading) for each individual covariate while holding all other covariates at their mean observed values. Parameter space for each covariate value (x-axis) is given for a realistic range of values. Various combinations with other variables (interaction or additive effect), discretized to factor values are given in plots with both solid and dashed lines.

The spatio-temporal components in the best model included the terms: CENTER, POSCOUNT (log transformed), NIGHTPROP, and the interactions of NIGHTPROP*CENTER and NIGHTPROP*POSCOUNT (Table 1). In general, the probability of a feeding event increased as the NIGHTPROP (Fig 2A) and POSCOUNT increased (Fig 2B). The interaction between these terms indicated that cases with a high POSCOUNT and a low NIGHTPROP were not as likely to represent a feeding event (Fig 2B), such as in the case of day-bed clusters that were used for extended periods. The CENTER covariate indicated that less spatially dispersed clusters were more likely to represent a feeding event than a cluster dispersed over a larger area (Fig 2C). However, the interaction between NIGHTPROP and CENTER indicated that a more dispersed cluster could also have a relatively high probability of representing a feeding event if the proportion of GPS locations recorded at night was relatively low. This situation may occur when a cougar day-beds away from the carcass it is feeding on, but within the 200 m spatial window defined by the clustering algorithm.

### Activity and Ground-truthing Covariates

Using the activity covariates alone in a model, an AUC measure of 0.819 was found, which rivalled models with spatio-temporal measures alone (AUC: 0.823) (Table 2). Overall, models including a combination of spatio-temporal and activity covariates were superior to ones without this combination, based on much lower AIC scores (delta AIC: > 133) and an approximate six percentage point increase in the predictive performance measures (Table 2). The activity grouping, included the x-axis average measure (ACCX) along with its quadratic form (ACCX^2^) indicated a general positive concave downward relationship between feeding probability and an increase in activity on the x-axis (increased forward-backward movements) (Fig 2F). A similar relationship was found when testing the y-axis average activity measure (ACCY). The ACCY relationship was not as strong as the ACCX relationship and showed a significant positive correlation with ACCX (Pearsons correlation = 0.934); thus ACCY was removed. ACCXYDIFF indicated that as the difference between x- and y-axis became more positive (x-axis measures stronger than y-axis measures), the probability of a feeding event increased, while a more negative difference (y-axis measures stronger than x-axis measures), indicated a decreased probability of a feeding event (Fig 2G). Two interactions were important in models using activity and spatio-temporal terms. The ACCX*CENTER interaction was indicative of tightly grouped clusters having a higher feeding probability when activity was higher, and a lower probability when activity was lower (Fig 2I). The ACCXYDIFF*POSCOUNT term allowed clusters with a relatively large number of GPS locations to not represent a feeding site in the case where y-axis movements dominated over x-axis movements (more negative difference) (Fig 2H), which may occur where a cougar is making repeated travelling movements in a restricted area over a longer time period without feeding.

Adding the ground-truthing error covariates, model fit via AIC did improve, but showed no considerable improvement in predictive performance measures. As the proportion of GPS locations available to the ground-truthing investigator increased (FIELDPROP), the probability of a feeding event generally increased, with the effect diminishing at higher proportions, as indicated by the quadratic form (FIELDPROP^2^) (Fig 2D). As SEARCH_LAG increased, the probability of a feeding event did not decrease curvilinearly as predicted, but decreased as a simple linear function of time between the cougar and ground-truthing visitation (Fig 2E). FIXRATE did show up as an important variable when considering models without the activity covariate, but was less important once seasonality was considered. FIXRATE eventually proved to be unimportant once considering models with activity measures. Not correcting for the ground-truthing errors when making predictions of the count of feeding events resulted in a 9.3% decline in the number of clusters predicted to be feeding events by the top model (Table 2).

Adding the SEAS covariate, lower AIC scores were achieved along with slightly improved prediction performance measures. However, this seasonal effect was only present in the summer season. Considering SEAS as a dummy variable specific to the summer season; the probability of a feeding event increased. Removing the SEAS covariate from the best model, performance measures of 0.881 were maintained (Table 2).

### Double Observer Ground-truthing Experiment

Standard visits were carried out on 174 clusters visited prior by an initial cluster observer. Feeding events on large prey (i.e., adult ungulates) made up 65.5% of these clusters. Small ungulate prey items (i.e., fawns) and non-ungulates (i.e., meso-carnivores) made up 16.1% and 12.1% respectively. No prey remains were found in 6.3% of the cases. Observers were non-concordant on the presence of prey remains in six or 3.5% of the cases. Of these six, two were when the standard ground-truthing observers misclassified the presence of prey remains, one of which was of a small prey item. Surprisingly, the remaining four cases were of the initial observer missing prey remains that the standard ground-truthing observer did find. Of these four feeding events misclassified by the initial investigator, one was a large prey item, while the remaining three were small or non-ungulate prey.

## Discussion

The results demonstrate that modeling the presence/absence of prey remains from ground-truthed GPS cluster investigations can be improved by including other biotelemetry measures in addition to the standard spatio-temporal characteristics of the GPS locational data. Like previous studies, we show that ground-truthing errors can influence the feeding prediction model, but the degree that errors influenced model predictions had not yet been measured. Furthermore, we show experimentally that ground-truthing visits conducted with a search lag of 2–60 days can be highly accurate at determining presence/absence of feeding activities.

The most parsimonious feeding prediction model achieved approximately a 90% AUC. Given that sensitivity/specificity was balanced when choosing a cut-off probability, this corresponded to 90% accuracy (proportion successfully classified). Ground-truthing input utilized all evidence of feeding remains as model input, regardless of prey size. Other previous cougar studies only modeled the visitations that yielded larger prey remains (> 8–15 kg; [1,11,12]). Our study was able to use a wider range of prey sizes, despite having GPS acquisition rates and ground-truthing methods similar to that of other studies [1,11–13]. However, making comparisons across studies is difficult, as the methods of choosing the cut-off probability are not consistent.

Season, habitat, and biological characteristics of the collared animal have also been incorporated in previously published models [31]. While potentially independent of spatio-temporal data, including these may yield a less generalizable prediction model. Parameterizing a model to include an individual animal effect (i.e., via mixed effects model) [31], may also result in a less general model. A more general model would be helpful in the instance where a few telemetered subjects were not accessible to ground-truthing observers; including random effects would not allow predictions to be made easily to these subjects. We do recognize that including random effect terms in a GLM may influence the relationships and coefficients estimated. To test whether an individual effect was present in our data set, we ran all models with the individual identifier as a random intercept term (R ‘lme4’ package). However, no improvement in parsimony or prediction performance was found (AIC > 709.1, AUC < 0.883).

### Activity Complementation

The largest improvement to our model came from implementing aggregated activity measures from the activity sensors as another covariate. The best model utilizing activity covariates alone had prediction performance measures rivaling that of models using only standard spatio-temporal covariates. When used in conjunction with spatio-temporal covariates, the activity covariate provided information on the relative degree of non-resting behavior the cougar was engaged in. While spatio-temporal covariates may indicate whether a predator is stationary or not, activity sensor data helps shed light on whether the stationary predator is engaged in a relatively non-active (i.e., animal is resting more) or active (i.e., animal is potentially feeding) state. Including these spatio-temporal and activity variables in a single GLM allowed us to test and confirm the various interactions between these independent predictors.

We used dual axis activity sensors that produced two measures that were generally correlated. Previous authors noted this attribute [32] with some authors subsequently discarding data from one axis [18]. However, dismissing an axis is not always a good idea, as shown in our analysis with ACCXYDIFF. While actual observational data indicating the types of behaviors leading to higher x- or y-axis activity measures is unavailable, we presume that feeding behaviors can be characterized by relatively more x-axis (forward and backward) motions, while travelling activities can be characterized by relatively more y-axis (side to side) motions. Subtracting y-axis quantities from x-axis quantities seem to have yielded more positive values while feeding and more negative values while not feeding. Future studies using direct observations of feeding and travelling predators would be able to confirm this.

Banfield [19] used cluster analysis and activity sensors to identify fine scale spatial and temporal characteristics of cougar hunting behaviors using a relatively high GPS acquisition rate of 15 minutes. He did not use the spatio-temporal and activity characteristics in the same model, but did initially identify kill sites using GPS spatio-temporal characteristics to define potential kills. Our study also relied on initially identifying potential feeding sites with the GPS location data, and thus our analysis is limited to the temporal resolution of our GPS fix rate and the scale of the identified GPS cluster. Raw activity data were available at approximately 5 minute intervals, but later aggregated to 3 to 4 hour intervals to more closely match that of the GPS fix rate, and then aggregated again to match that of the time spent at the cluster. This was a very coarse assessment of activity that can be improved upon in future studies, especially if shorter GPS acquisition intervals were available. Unfortunately, decreasing the GPS location acquisition interval to a very fine scale (i.e., 15 minutes [19]) would have a greater toll on battery life; reducing the time span an animal can be monitored. Expected battery life for collars placed on the cougars was approximately 1–2 years, which helped minimize animal recaptures over the course of the study. Reducing collar burden on smaller predators would also come with the cost of shortening battery life.

Considering raw activity data was available at 5 min intervals, further analysis conducted independently of the GPS locational data could reveal further improvements in the identification of feeding events on small prey. Future work may also benefit by incorporating measures from newer technologies, such as high-frequency (16 hz) accelerometer logging, especially if more detailed movements regarding resting and attack behaviors are of interest [33,34]. However, the coarse-scale accelerometer activity sensor and the simple data analysis demonstrated in our study are readily available to most practitioners. Our “accelerometer only” model (Table 1: ACVTY) was able to predict feeding activities with reasonable accuracy (>80%), which has not yet been demonstrated in similar cougar studies using fine-scale accelerometer data [34]. Future work should focus on methods that fully integrate both GPS location and activity data in a single processing step aimed at identifying the timing and spatial locations of behavior, rather than initially identifying events based on GPS location data alone ([19], this study) or the activity data alone [20,22]. Then, ground-truthing efforts in the field can focus on gathering sufficient callibration input from both GPS and biotelemetry data.

### Ground-truthing Errors

Like uni- and multiple regression analysis done in several past studies [23,24,35], SEARCH_LAG was an important predictor for the probability of feeding site presence. Examining the effect of search lag as a negative exponentially decreasing function showed no improvement in fit over a linear one. FIELDPROP indicated that there was a negative linear relationship, which was induced by imperfect satellite data retrieval methods using our GlobalStar communication system. Having all GPS locations available to ground-truthing observers is important for small prey remains that result in clusters of fewer GPS locations [1, 12, 23], which are more sensitive to having an inaccurately defined search area where observers focus their efforts. FIXRATE did have an effect in simpler models, but was likely absorbed by the effect of other covariates after expanding to more complex models. Accounting for any effect of fix acquisition rate would be difficult, as an imperfect fix rate can result in clusters not even being established. Thus it is advised that future studies afflicted with low fix success rates use shorter GPS location acquisition intervals (if battery life is permitting) to help avoid such biases. Shorter intervals would likely yield more GPS locations being available as “backup” in the case of short prey handling times. Additionally, shorter fix acquisition intervals should yield improved fix acquisition success [36].

We included models with SEAS as a predictor, which may not have a sole association with degradation of prey remains, but with the underlying prey availability. Availability of prey, whether temporally or spatially varying, may influence the overall proportion of clusters that are truly feeding events. This may be especially true in the summer season when cougars appeared to shift to feeding on smaller prey more frequently [37]. It is important to note that our robust temporally stratified sampling design was critical for assessing and accounting for the effect of ground-truthing error covariates, as applying a more opportunistic sampling scheme would potentially confound SEARCH_LAG with other covariates.

This study is the first to actually account for ground-truthing errors in final estimates of kill counts/rates, which resulted in an approximately 10% increase in the number of clusters to be predicted as feeding events. Ultimately, this can influence important predation parameters such as kill-rates [1,12]. Setting the SEARCH_LAG and FIELDPROP variables in the entire dataset (ground-truthed and non-ground-truthed sites) to a value of 0 and 1 respectively, made the assumption that our logistic regression model was created by an adequate sample of observations near these extreme values (SEARCH_LAG ≤ 5 days). Failing to meet this assumption may risk having fitted values at these extremes to be determined by highly influential/high leverage observations. While not shown, we did explore whether bolstering the sample of clusters investigated with a SEARCH_LAG of 0–5 days with initial investigation clusters (Fig 1) would change the response of the SEARCH_LAG covariate. Finding no effect with this addition, we feel that the assumption was met.

The experimental test using the double blind observers indicated that a very small proportion (3.5%) of feeding sites may go undetected by an observer visiting 2–60 days post mortem of the prey animal. This initially appeared contradictory to the response shown for SEARCH_LAG and FIELD_PROP covariates in the prediction logistic regression model. However, comparing the number of predicted feeding events using the corrected method (3360 predicted) to the uncorrected method (3040 predicted) it appears that only 9.3% of the events are missed as a result of ground-truthing errors. Upon closer inspection of the experimental data, 4 of the 6 sites misclassified by at least one observer were of small prey items, despite large prey items making up 66% of the feeding events found at all clusters visited twice. This supports suggestions from other studies that most misclassified feeding events are of smaller prey items [12,13,38].

Four of the six false-absence cases when classifying prey presence were when the initial investigation failed to uncover prey remains that were eventually found by the standard investigator. We believe this is because initial ground-truthing observers often visited the cluster without all GPS locations in hand. Additionally, the initial observer must avoid disturbing the cougar while it fed, leading to a less thorough search effort. In these cases of misclassification by the initial observer, the investigation was conducted with only the first two GPS locations of the cluster in hand and therefore was unable to locate prey remains that were likely just out of the search buffer. This was supported by the observational logistic regression model results indicating that the proportion of GPS locations an investigator has in hand (FIELDPROP) when visiting the cluster can influence the probability of finding a feeding event. Future GPS cluster studies using remote data retrieval methods, such as GlobalStar or Iridium satellites, should be aware of this potential issue and make attempts to obtain all GPS location data in hand, constituting the cluster, to direct the search effort.

Visiting a potential feeding site soon after a suspected kill is often the only way mortality can be truly determined, especially considering the role that scavenging may play in the diets of many predators (e.g., cougar; [39–41]). Visiting the kill while the edible material is still intact on the carcass is usually necessary for determining sign left by the predator that ultimately killed the prey. Visiting the kill any later, evidence may be consumed. However, this can lead to other problems related to finding prey remains and the ethics of potentially disturbing the predator. In approximately 25% of the initial ground-truthing visits, the cougar was encountered by the observer near the cluster. In most cases, the cougar stayed on site within a short distance of the prey carcass. If the cougar was displaced by the observer, it returned shortly after or later in the evening. While we did suspect a few instances of prey abandonment, it was uncertain that these were directly attributable to observer disturbance.

## Conclusion

We provide future researchers studying carnivore feeding behaviors with some readily available improvements. We demonstrate how other biotelemetry data sources (i.e., activity sensors) can be easily integrated into the commonly used logistic regression GLM. Future studies incorporating activity sensor data into a GLM along with GPS spatio-temporal data will benefit by reducing GPS location acquisition rates to more closely match the fine time scale of the activity sensor data. However, practitioners must weigh the cost of reducing GPS collar battery life when making such reductions.

The experimental double-observer study showed that ground-truthing only misses a small percentage (~4%) of feeding events, even with a mean search delay lag of 30 days. The statistical observation model indicated that any missed feeding events are a function of visitation lag and the amount of spatial information available to ground-truthing observers. However, correcting prediction models to account for this source of error, a small increase in the number of feeding events resulted. This increase supports the findings of the experimental study. The methods employed in this study for standard ground-truthing visits followed that of the pioneering studies in similar systems using cougars as a model species, thus we do not believe this error to be completely unique. Therefore, past studies using GPS cluster techniques to estimate parameters (i.e., kill rates), may have produced parameters that were biased low. As sensor technology improves, the amount and detail of information gathered will only improve prediction models. However, the need to create a model training data set will still be present, whether done with ground-truthing visitations by human observers or by other means. Practitioners should be aware of false-absence sources in ground-truthing and the degree at which these errors may affect behavior prediction models.

## Supporting Information

S1 Dataset Standard ground-truthing visitation input dataset. This dataset is a table of the unique clusters visited by the standard ground-truthing observer. This is used as the input data set for conducting the logistic regression analysis for predicting feeding sites. Independent variable field names are intuitive to the variable naming used in the manuscript. The dependent variable (binary response variable) is denoted as “FEEDING”.
(CSV)

S2 Dataset Clusters visited and non-visited. This dataset is a table of the unique clusters (visited and non-visited combined) of GPS locations created the cougars. This is required for deriving the predicted feeding event counts. Field names are intuitive to the variable names used in the manuscript.
(CSV)

S3 Dataset Double observer ground-truthing input dataset. This dataset is used for the Double Observer Ground-truthing Experiment. LargeSizedPrey = binary indicator for whether prey found was a large sized animal (>23.7kg), WildUngulate = binary indicator for whether prey found was a mule deer or elk, Visit1FEEDING = binary indicates whether the initial ground-truthing observer found prey remains, Visit1SEARCH_LAG = number of days passed between the start of the cluster and when the initial ground-truthing observer visited the site, Visit2FEEDING = binary indicates whether the second (standard) ground-truthing observer found prey remains, Visit2SEARCH_LAG = number of days passed between the start of the cluster and when the second (standard) ground-truthing observer visited the site.
(CSV)

## References

[1] Anderson C, R, Lindzey FG (2006) Estimating cougar predation rates from GPS location clusters. J Wildl Manage 67:307–316.

[2] Burkholder BL (1959) Movements and behavior of a wolf pack in Alaska. J Wildl Manage 23:1–11.

[3] Ross IP, Jalkotzy MG (1996) Cougar predation on moose in southwestern Alberta. Alces 32:1–8.

[4] Hebblewhite M, Paquet PC, Pletscher DH, Lessard RB, Callaghan CJ (2003) Development and application of a ratio estimator to estimate wolf kill rates and variance in a multiple-prey system. Wildl Soc Bull 31:933–946.

[5] Balme G, Hunter L, Slotow R (2007) Feeding habitat selection by hunting leopards (Panthera pardus) in a woodland savanna: prey catchability versus abundance. Anim Behav 74:589–598. 10.1016/j.anbehav.2006.12.014

[6] Rieth WR (2010) Cougar resource selection in two mountain ranges in Utah: a study on scale and behavior. Logan, Utah, USA, Utah State University.

[7] Zeller KA, McGarigal K, Beier P, Cushman SA, Vickers TW, Boyce WM (2014) Sensitivity of landscape resistance estimates based on point selection functions to scale and behavioral state: pumas as a case study. Landscape Ecol 29:541–557. 10.1007/s10980-014-9991-4

[8] Hopcraft JGC, Sinclair ARE, Packer C (2005) Planning for success: Serengeti lions seek prey accessibility rather than abundance. J Anim Ecol 74:559–566. 10.1111/j.1365-2656.2005.00955.x

[9] Laundré JW (2010) Behavioral response races, predator-prey shell games, ecology of fear, and patch use of pumas and their ungulate prey. Ecology 91:2995–3007. 10.1890/08-2345.1 21058559

[10] Davidson Z, Valeix M, Loveridge AJ, Hunt JE, Johnson PJ, Madzikanda H, et al (2012) Environmental determinants of habitat and kill site selection in a large carnivore: scale matters. J Mammal 93:677–685. 10.1644/10-MAMM-A-424.1

[11] Wilmers CC, Wang Y, Nickel B, Houghtaling P, Shakeri Y, Allen ML, et al (2013) Scale dependent behavioral responses to human development by a large predator, the puma. PLoS One 8:e60590 10.1371/journal.pone.0060590 23613732 PMC3629074

[12] Knopff KH, Knopff AA, Warren MB, Boyce MS (2009) Evaluating Global Positioning System Telemetry Techniques for Estimating Cougar Predation Parameters. J Wildl Manage 73:586–597. 10.2193/2008-294

[13] Ruth TK, Buotte PC, Quigley HB (2010) Comparing Ground Telemetry and Global Positioning System Methods to Determine Cougar Kill Rates. J Wildl Manage 74:1122–1133. 10.2193/2009-058

[14] Webb NF, Hebblewhite M, Merrill EH (2008) Statistical Methods for Identifying Wolf Kill Sites Using Global Positioning System Locations. J Wildl Manage 72:798–807. 10.2193/2006-566

[15] Rauset GR, Kindberg J, Swenson JE (2012) Modeling female brown bear kill rates on moose calves using global positioning satellite data. J Wildl Manage 76:1597–1606. 10.1002/jwmg.452

[16] Miller CS, Hebblewhite M, Petrunenko YK, Seryodkin IV, DeCesare NJ, Goodrich JM, et al (2013) Estimating Amur tiger (Panthera tigris altaica) kill rates and potential consumption rates using global positioning system collars. J Mammal 94:845–855. 10.1644/12-MAMM-A-209.1

[17] Fröhlich M, Berger A, Kramer-Schadt S, Heckmann I, Martins Q (2012) Complementing GPS Cluster Analysis with Activity Data for Studies of Leopard (Panthera pardus) Diet. S. Afr. J Wildl Res 42:104–110.

[18] Podolski I, Belotti E, Bufka L, Reulen H, Heurich M (2013) Seasonal and daily activity patterns of free-living Eurasian lynxLynx lynxin relation to availability of kills. Wildl Biol 19:69–77. 10.2981/12-049

[19] Banfield JE (2012) Cougar response to roads and predatory behaviour in southwestern Alberta, University of Alberta.

[20] Grunewalder S, Broekhuis F, Macdonald DW, Wilson AM, McNutt JW, Shawe-Taylor J, et al (2012) Movement activity based classification of animal behaviour with an application to data from cheetah (Acinonyx jubatus). PLoS One 7:e49120 10.1371/journal.pone.0049120 23185301 PMC3501513

[21] McClintock BT, Russell DJF, Matthiopoulos J, King R (2012) Combining individual animal movement and ancillary biotelemetry data to investigate population-level activity budgets. Ecology 94:838–849. 10.1890/12-0954.1

[22] Gervasi V, Brunberg S, Swenson JE, Bowman J (2006) An Individual-Based Method to Measure Animal Activity Levels: A Test on Brown Bears. Wildl Soc Bull 34:1314–1319. 10.2193/0091-7648(2006)34[1314:AIMTMA]2.0.CO;2

[23] Tambling CJ, Cameron EZ, Du Toit JT, Getz WM (2010) Methods for Locating African Lion Kills Using Global Positioning System Movement Data. J Wildl Manage 74:549–556. 10.2193/2009-010

[24] Svoboda NJ, Belant JL, Beyer DE, Duquette JF, Martin JA (2013) Identifying bobcat (Lynx rufus) kill sites using a global positioning system. Wildl Biol 19:78–86. 10.2981/12-031

[25] Alldredge MW, Bergman EJ, Bishop C, Logan KA, Freddy DJ (2008) Pilot evaluation of predator-prey dynamics on the Uncompahgre Plateau WP 3001: Wildlife Research Report, Mammals Program. Fort Collins, CO: Colorado Parks and Wildlife.

[26] Armstrong DM, Fitzgerald JP, Meaney CA (2011) Mammals of Colorado 2nd edition Denver Museum of Nature and Science. Boulder, CO: University Press of Colorado.

[27] Anderson AE, Medin DM, Bowden DC (1974) Growth and morphometry of mule deer carcass, selected bones, organs, and glands of Mule Deer. Wildlife Monographs 39:9–122.

[28] Burnham KP, Anderson DR (2002) Model selection and model inference: a practical information-theoretic approach. 2nd edition ed. New York, NY, USA: Springer-Verlag.

[29] Hosmer DW, Lemeshow S (2000) Applied Logistic Regression. 2nd edition ed. New York, NY, USA: John Wiley and Sons, Inc.

[30] Boyce MS, Vernier PR, Nielsen SE, Schmiegelow FKA (2002) Evaluating resource selection functions. Ecol Model 157:281–300. 10.1016/S0304-3800(02)00200-4

[31] Smith JA, Wang Y, Wilmers CC (2015) Top carnivores increase their kill rates on prey as a response to human-induced fear. Proc R Soc Lond [Biol] 282(1802). 10.1098/rspb.2014.2711 PMC4344154 25608884

[32] Löttker P, Rummel A, Traube M, Stache A, Šustr P, Müller J, et al (2009) New possibilities of observing animal behaviour from a distance using activity sensors in GPS-collars. Wildl Biol 15:425–434. 10.2981/08-014

[33] Williams TM, Wolfe LL, Davis T, Kendall T, Richter B, Wang Y, et al (2014) Mammalian energetics. Instantaneous energetics of puma kills reveal advantage of felid sneak attacks. Science 346(6205):81–85. 10.1126/science.1254885 25278610

[34] Wang Y, Nickel B, Rutishauser M, Bryce C, Williams TM, Ekaim G, et al (2015) Movement, resting, and attack behaviors of wild pumas are revealed by tri-axial accelerometer measurements. Movement Ecology 3:2 10.1186/s40462-015-0030-0 25709837 PMC4337468

[35] Elbroch LM, Wittmer HU (2013) The effects of puma prey selection and specialization on less abundant prey in Patagonia. J Mammal 94:259–268. 10.1644/12-MAMM-A-041.1

[36] Cain JW III, Krausman PR, Jansen BD, Morgart JR (2005) Influence of topography and GPS fix interval on GPS collar performance. Wildl Soc Bull 33:926–934. 10.2193/0091-7648(2005)33[926:IOTAGF]2.0.CO;2

[37] Knopff KH, Knopff AA, Kortello A, Boyce MS (2010) Cougar Kill Rate and Prey Composition in a Multiprey System. J Wildl Manage 74:1435–1447. 10.2193/2009-314

[38] Bacon MM, Becic GM, Epp MT, Boyce MS (2011) Do GPS clusters really work? carnivore diet from scat analysis and GPS telemetry methods. Wildl Soc Bull 35:409–415. 10.1002/wsb.85

[39] Bauer JW, Logan KA, Sweanor LL, Boyce WM (2005) Scavenging Behavior in Puma. The Southwestern Naturalist 4:466–471. 10.1894/0038-4909(2005)050[0466:SBIP]2.0.CO;2

[40] Bacon MM, Boyce MS (2010) Scavenging of an Elk, Cervus elaphus, carcass by multiple cougars (Puma concolor), in southeastern Alberta. The Canadian Field-Naturalist 124:242–245.

[41] Knopff KH, Knopff AA, Boyce MS (2010) Scavenging Makes Cougars Susceptible to Snaring at Wolf Bait Stations. J Wildl Manage 74:644–653. 10.2193/2009-252

